# Analysis of microsatellites from the transcriptome of downy mildew pathogens and their application for characterization of *Pseudoperonospora* populations

**DOI:** 10.7717/peerj.3266

**Published:** 2017-05-02

**Authors:** Emma C. Wallace, Lina M. Quesada-Ocampo

**Affiliations:** Department of Plant Pathology, North Carolina State University, Raleigh, NC, United States

**Keywords:** *Pseudoperonospora cubensis*, Downy mildew, Simple Sequence Repeats, Microsatellites, Comparative genomics, Population genetics

## Abstract

Downy mildew pathogens affect several economically important crops worldwide but, due to their obligate nature, few genetic resources are available for genomic and population analyses. Draft genomes for emergent downy mildew pathogens such as the oomycete *Pseudoperonospora cubensis*, causal agent of cucurbit downy mildew, have been published and can be used to perform comparative genomic analysis and develop tools such as microsatellites to characterize pathogen population structure. We used bioinformatics to identify 2,738 microsatellites in the *P. cubensis* predicted transcriptome and evaluate them for transferability to the hop downy mildew pathogen, *Pseudoperonospora humuli*, since no draft genome is available for this species. We also compared the microsatellite repertoire of *P. cubensis* to that of the model organism *Hyaloperonospora arabidopsidis*, which causes downy mildew in Arabidopsis. Although trends in frequency of motif-type were similar, the percentage of SSRs identified from *P. cubensis* transcripts differed significantly from *H. arabidopsidis*. The majority of a subset of microsatellites selected for laboratory validation (92%) produced a product in *P. cubensis* isolates, and 83 microsatellites demonstrated transferability to *P. humuli*. Eleven microsatellites were found to be polymorphic and consistently amplified in *P. cubensis* isolates. Analysis of *Pseudoperonospora* isolates from diverse hosts and locations revealed higher diversity in *P. cubensis* compared to *P. humuli* isolates. These microsatellites will be useful in efforts to better understand relationships within *Pseudoperonospora* species and *P. cubensis* on a population level.

## Introduction

Downy mildew pathogens are obligate oomycetes that cause devastating epidemics worldwide in food, ornamental, and landscape plant species. In recent years, draft genomes of emergent downy mildew pathogens have been published such as the cucurbit downy mildew pathogen *Pseudoperonospora cubensis* and the sunflower downy mildew pathogen *Plasmopara halstedii* (Savory et al., 2012b; Sharma et al., 2015). Nonetheless, several genera of economically important downy mildew pathogens such as *Pseudoperonospora humuli*, *Plasmopara obducens*, *Peronospora belbahrii*, and *Plasmopara viticola*, which cause downy mildew of hop, impatiens, basil, and grape respectively (Lebeda & Cohen, 2011; Gent et al., 2009; Ristic et al., 2011; Kanetis et al., 2013; Gessler, Pertot & Perazzolli, 2011) have few or no genomic resources available.

In the case of *P. cubensis,* which exhibits population stratification by host in the United States, methods to easily determine population structure in a particular region are crucial to understand disease epidemiology and develop effective and durable management strategies (Quesada-Ocampo, Granke & Olsen, 2012). Prior to 2004, losses in cucumber due to *P. cubensis* were minimal and were managed by host resistance. After the emergence of a highly virulent strain, fungicides are now a necessity to control this pathogen (Colucci, Wehner & Holmes, 2006; Holmes et al., 2015). The prolific asexual sporulation of the pathogen on the leaf surface and the ease through which it is aerially dispersed from field to field also contribute to the pathogen’s damaging effects. Although the role of sexual reproduction and wild cucurbit hosts as inoculum sources still have not been thoroughly determined, epidemiological studies have helped develop control strategies (Lebeda & Cohen, 2011; Cohen et al., 2015; Holmes et al., 2015; Ojiambo et al., 2015; Wallace et al., 2014; Wallace, Adams & Quesada-Ocampo, 2015). To date, *P. cubensis* continues to cause major losses as vital fungicides lose efficacy (Ojiambo et al., 2015; Holmes et al., 2015). Similarly, *P. humuli* outbreaks have increased in the United States in recent years due to the expansion of the hop industry in the East coast and now the geographic range of *P. cubensis* overlaps that of *P. humuli*.

Several studies have investigated the phylogenetic relationships between *P. cubensis* and *P. humuli*. In 2005, researchers suggested there were no real differences in morphology between *P. cubensis* and *P. humuli*. They also concluded that based on the genetic similarity of nrITS sequences, *P. humuli* is the same species as *P. cubensis* (Choi, Hong & Shin, 2005). Nonetheless, results from studies using more genetic markers support the species distinction between *P. humuli* and *P. cubensis* with nrITS, *coxII*, and *ypt1* (Runge, Choi & Thines, 2011)*.* Furthermore, the idea of two distinct species was supported through phylogenetic analysis of nrITS, *B-tub*, and *cox2* (Mitchell et al., 2011). However, it was found that in laboratory settings, *P. cubensis* can infect hop and have light sporulation and that *P. humuli* can infect cucumber with limited success (Mitchell et al., 2011; Runge & Thines, 2012). Due to the potential cross infectivity of *P. cubensis* and *P. humuli*, overlapping geographical range, and genetic similarity, using markers such as microsatellites that allow resolving population structure among these closely related species is desirable.

Microsatellites, or Simple Sequence Repeats (SSRs) are repeating base pair units consisting of 1–6 nucleotide sequences. They occur frequently throughout the genome and have high mutation rates (10^−3^–10^−4^ per locus per generation) resulting in differences in the number of repeated motifs at particular loci between certain individuals. Before the prevalence and accessibility of genomic resources and high-throughput sequencing technology, microsatellite identification was an expensive and laborious process. *De novo* identification involved creating a genomic library, identifying SSR-containing clones, sequencing, primer design, and laboratory validation, which was a labor-intensive and expensive process (Abdelkrim et al., 2009; Zane, Bargelloni & Patarnello, 2002). Now SSR identification and marker development can be streamlined with next generation sequencing and bioinformatics tools. Microsatellites within genes are useful as they provide functional information about the marker, tend to be conserved, and are transferable between species (Liu et al., 2013). There have been few attempts at developing microsatellites for *P. cubensis,* the microsatellite repertoire has not been compared to that of sequenced downy mildew pathogens (Garnica et al., 2006), the markers have not been used in an extensive population study to date (Almany et al., 2009; Hadziabdic et al., 2013), or have been tested for transferability to *P. humuli* (Naegele et al., 2014; Naegele et al., 2016).

In light of the economic importance of *P. cubensis* and *P. humuli,* and the potential insight offered through the use of microsatellites for population analysis, we sought to: (1) Analyze and compare the microsatellite repertoire in the *P. cubensis* and *Hyaloperonospora arabidopsidis* predicted transcriptomes; (2) Evaluate microsatellites for use on *P. cubensis* population analyses and determine species transferability to *P. humuli*; and (3) Characterize the population structure of *Pseudoperonospora* isolates from a wide host range and several geographic regions using microsatellite fragment analysis. Overall, in this study we demonstrated that *in silico* identification of microsatellites in predicted genes from *P. cubensis* next generation sequencing data provides a substantial amount of informative markers that can be transferred to closely related downy mildew pathogens and used in population structure analyses.

## Materials and Methods

### *In silico* identification and analyses of SSRs in predicted downy mildew transcriptomes

Transcriptomes predicted from genome assemblies for two downy mildew pathogens, *Pseudoperonospora cubensis* and *Hyaloperonospora arabidopsidis* that are publicly available were used for SSR identification. The FASTA file for the *P. cubensis* genome sequences and the .gff3 file with predicted gene coordinates were downloaded from Savory et al. (2012a) and Bioperl was used to generate a predicted transcriptome (Fig. S1). The genome assembly of *H. arabidopsidis* is described in Baxter et al. (2010), and is located in EMBL/Genbank/DDBJ databases under accession GCA_000173235.2. The transcripts predicted from the genome assembly were downloaded from EnsmblProtists database under HyaAraEmoy2_2.0.

The Microsatellite Identification Tool (MISA) (Thiel et al., 2003) was used to search the *P. cubensis* and *H. arabidopsidis* transcriptomes for the presence of microsatellites. MISA reported perfect and compound microsatellites ranging from one to six base pair units with a specified minimum number of repeats for each motif, specifically, 1/20, 2/5, 3/4, 4/3, 5/3, 6/3 (unit size/minimum number of repeats). Comparisons between microsatellite abundance, frequency, and motif types in the two downy mildew transcriptomes were calculated with a proportion test as conducted in Garnica et al. (2006).

### Primer design

Bioperl programing was used to parse the MISA output file so the program Primer3 (Rozen & Skaletsky, 2000) could use sequence coordinates reported by MISA to design primers flanking the identified microsatellites. The program designed primers that would amplify products between 100 and 300 bp. The primers were to be between 18 and 27 bp with optimum length of 20 bp, with GC content between 20% and 80% with optimum GC content of 50%, and with a melting temperature between 57 and 63 °C with the optimum melting temperature of 60 °C. Primers representative of the different motif groups (100 total) were ordered from and manufactured by Integrated DNA Technologies, Inc. (Coralville, IA, USA). Forward primers were designed to include an M13 tail for fluorescent labeling of products and later fragment analyses (Schuelke, 2000).

### Tissue collection and DNA extraction

A total of 11 *Pseudoperonospora cubensis* isolates and two *P. humuli* isolates were used to evaluate 100 Primer3-generated microsatellite primers for amplification, transferability, and polymorphism via gel electrophoresis. For analysis with fragment analysis, 38 *P. cubensis* isolates and 22 *P. humuli* isolates were screened (Table 1). Cucurbit and hop leaves infected with downy mildew were collected from several locations throughout North Carolina (NC) in 2013 and 2014 (Lenoir, Haywood, Johnston, and Rowan counties). The presence of the pathogen on leaves was confirmed by the observation of sporulation using a dissecting microscope. Leaf lesions were excised by sterile scalpel, placed in a microfuge tube, and stored at −80 °C until the time of DNA extraction. The isolate used in the sequencing of the *P. cubensis* genome (MSU-1) was included as a positive control (Savory et al., 2012a; Savory et al., 2012b). Collaborators provided isolates from other geographic regions and DNA was extracted from pelleted sporangia (Table 1). Tissue was disrupted and DNA was extracted and purified via phenol-chloroform extractions adapted from previous work (Ahmed et al., 2009). DNA was purified with ethanol washes then suspended in 1× TE buffer and quantified using a NanoDrop ND 1000 spectrophotometer and NanoDrop 2.4.7c software (NanoDrop Technologies Inc., Wilmington, DE, USA). Integrity of the DNA was confirmed by gel electrophoresis with the presence of a >12,000 bp band. Each isolate was amplified in the nrITS and mitochondrial Nad1 and Nad5 (Quesada-Ocampo, Granke & Olsen, 2012) where presence of a band of appropriate size (700 bp, 500 bp, 300 bp, respectively) confirmed the presence of *P. cubensis* or *P. humuli* DNA in lesion tissue and a lack of a band confirmed negative controls (uninfected cucumber leaf tissue and water control).

### Primer evaluation with gel electrophoresis

The microsatellite primers report from the Primer3 output was divided by microsatellite motif types (tri-, tetra-, penta-, hexa-nucleotide repeats) and arranged from highest number of repeating units in descending order, as microsatellites with more repeats tend to be more polymorphic (Guichoux et al., 2011; Ellegren, 2000). The top fifteen primers of each motif type were selected to be validated for amplification and tested for consistency and transferability to *P. humuli* in a screen against the eleven *Pseudoperonospora cubensis* isolates from different cucurbit hosts (*Cucumis sativus*, *Cucumis melo*, *Cucurbita pepo*, *Cucurbita maxima*, *Cucurbita moschata*, *Citrullus lanatus*, *Momordica balsamina*, and *Momordica charantia*) and two *P. humuli* isolates (Table 1). Forward primers were labeled with a partial M13 tail (GACGGCCAGT) on the 5′ end so they could be used in fragment analysis downstream. Preliminary results suggested microsatellites with certain motifs were more likely to be polymorphic, so more markers with tri- and hexa-nucleotide repeats were evaluated. PCR reactions were performed with 10 µL of 2xGoTaq^®^ Hot Start Green Master Mix (Promega, Madison, WI, USA), 1 µL of 10 µM forward primer, 1 µL of 10 µM reverse primer, 10 ng of DNA, and sterile water. The thermal cycler (Bio-Rad, Hercules, CA) program, CDMSSR1, was set to have an initial denature of 94 °C for 3 m, and 35 repeating cycles consisting of denaturing at 94 °C, annealing at 53 °C, and extension at 72 °C, each step having a duration of 30 s. This program concluded with a 5 m final extension at 72 °C.

*P. cubensis* isolates from watermelon, bitter melon, and balsam apple, and the positive and negative controls were amplified by touchdown PCR (TDSSR1) because infected leaves with low levels of sporulation did not amplify reliably with standard PCR settings. These isolates underwent PCR with thermal cycler settings with an initial denaturing step of 94 °C for 5 m, then 20 cycles of a 30 s denaturing step at 94 °C, a 45 s at an annealing temperature starting at 62 °C, and an extension step at 72 °C for 2 m. At each cycle, the annealing temperature would decrease by 0.5 °C. Then the reaction continued with another twenty cycles of a 30 s denature step at 94 °C, a 45 s annealing step at 55 °C, and a 2 m extension step at 72 °C. The reaction ended with a 5 m final extension at 72 °C (Korbie & Mattick, 2008). These 13 *Pseudoperonospora* isolates were screened against a total of 100 SSR markers and evaluated via gel electrophoresis. PCR products were run on 4% ultrapure agarose gels at 40 volts for approximately five hours to evaluate amplification and determine product size.

### Fragment analysis of polymorphic SSR primers

From the initial screening of 100 microsatellite markers with agarose gel electrophoresis, a subset of 17 primers with consistent amplification across samples and appearance of polymorphism across *P. cubensis* isolates were further analyzed. These 17 primers were applied to 38 *P. cubensis* isolates and 22 *P. humuli* isolates and evaluated for polymorphism and consistency via fragment analysis (Table 1). PCR products of downy mildew isolates amplified with the polymorphic SSR primers were subjected to a second PCR, CDMSSR2, in order to attach fluorescent dyes to the amplified products. Reactions were carried out in 10 µL volume consisting of the same reagents and concentrations as above with the exception of an M13 primer (TGTAAAACGACGGCCAGT) tagged with a fluorescent dye in place of the site-specific forward primer (Schuelke, 2000). Thermal cycler settings for the program CDMSSR2 were the same as the CDMSSR1 program, but with 15 repeating cycles of denaturing, annealing, and extension steps. Products from the CDMSSR2 were diluted fifty to twenty-five-fold and pool-plexed, combining multiple PCR products of different microsatellite primers labeled with different fluorescent dyes (VIC and 6FAM) (Applied Biosystems, Foster City, CA, USA). A genotyping reaction was performed where HiDi Formamide, LIZ600 size standard, and the diluted, pool-plexed sample were combined then submitted to the NCSU Genomic Science Laboratory (GSL, Raleigh, NC, USA) for genotyping with a 3730xl DNA Analyzer (Applied Biosystems, Foster City, CA, USA).

**Table 1:** *Pseudoperonospora* isolates used for microsatellite screening.

**Isolate**	**Isolate species**	**Electrophoresis/ fragment analysis/both**	**Host (scientific name/variety)**	**Tissue used in DNA extraction**	**Isolate origin**	**Year collected**
KIN2-1-4	*P. cubensis*	Electrophoresis	*Cucumis sativus*, cv Straight 8	Infected leaf tissue	Lenoir County, NC	2013
WAY2-2A-1S	*P. cubensis*	Electrophoresis	*Cucumis sativus*, cv SVR14763462	Sporangia	Haywood County, NC	2013
Kin2-2a-4	*P. cubensis*	Fragment analysis	*Cucumis sativus*, cv SVR14763462	Infected leaf tissue	Lenoir County, NC	2013
14cle2-1-6a	*P. cubensis*	Fragment analysis	*Cucumis sativus*, cv Straight 8	Infected leaf tissue	Rowan County, NC	2014
14cle-1-7	*P. cubensis*	Fragment analysis	*Cucumis sativus*, cv Straight 8	Infected leaf tissue	Rowan County, NC	2014
14kin2-1-3B	*P. cubensis*	Fragment analysis	*Cucumis sativus*, cv Straight 8	Infected leaf tissue	Lenoir County, NC	2014
way2-3-1	*P. cubensis*	Both	*Cucumis melo*, cv Hales Best Jumbo	Infected leaf tissue	Haywood County, NC	2013
14kin2-3-2a	*P. cubensis*	Fragment analysis	*Cucumis melo*, cv Hales Best Jumbo	Infected leaf tissue	Lenoir County, NC	2014
14cle2-3-6a	*P. cubensis*	Fragment analysis	*Cucumis melo*, cv Hales Best Jumbo	Infected leaf tissue	Rowan County, NC	2014
cle2-3-10	*P. cubensis*	Fragment analysis	*Cucumis melo*, cv Hales Best Jumbo	Infected leaf tissue	Rowan County, NC	2013
way2-4-3	*P. cubensis*	Both	*Cucurbita pepo*, cv Table Ace	Infected leaf tissue	Haywood County, NC	2013
14cle2-4-3a	*P. cubensis*	Fragment analysis	*Cucurbita pepo*, cv Table Ace	Infected leaf tissue	Rowan County, NC	2014
cle2-4-8	*P. cubensis*	Fragment analysis	*Cucurbita pepo*, cv Table Ace	Infected leaf tissue	Rowan County, NC	2013
way2-4-7	*P. cubensis*	Fragment analysis	*Cucurbita pepo*, cv Table Ace	Infected leaf tissue	Haywood County, NC	2013
CLAY5_2	*P. cubensis*	Electrophoresis	*Cucurbita maxima*, cv Big Max	Infected leaf tissue	Johnston County, NC	2013
cle-5-7	*P. cubensis*	Fragment analysis	*Cucurbita maxima*, cv Big Max	Infected leaf tissue	Rowan County, NC	2013
14kin2-5-5a	*P. cubensis*	Fragment analysis	*Cucurbita maxima*, cv Big Max	Infected leaf tissue	Lenoir County, NC	2014
kin-5-4	*P. cubensis*	Fragment analysis	*Cucurbita maxima*, cv Big Max	Infected leaf tissue	Lenoir County, NC	2013
kin-5-9	*P. cubensis*	Fragment analysis	*Cucurbita maxima*, cv Big Max	Infected leaf tissue	Lenoir County, NC	2013
way2-6-2	*P. cubensis*	Both	*Cucurbita moschata*, cv Waltham butternut	Infected leaf tissue	Haywood County, NC	2013
14cle2-6-8a	*P. cubensis*	Fragment analysis	*Cucurbita moschata*, cv Waltham butternut	Infected leaf tissue	Rowan County, NC	2014
14kin-6-6	*P. cubensis*	Fragment analysis	*Cucurbita moschata*, cv Waltham butternut	Infected leaf tissue	Lenoir County, NC	2014
cle2-6-6	*P. cubensis*	Fragment analysis	*Cucurbita moschata*, cv Waltham butternut	Infected leaf tissue	Rowan County, NC	2013
CLE2-7-3	*P. cubensis*	Electrophoresis	*Cirtullus lanatus*, Micky Lee	Infected leaf tissue	Rowan County, NC	2013
cle-7-3	*P. cubensis*	Fragment analysis	*Citrullus lanatus*, Micky Lee	Infected leaf tissue	Rowan County, NC	2013
cle2-7-12	*P. cubensis*	Fragment analysis	*Citrullus lanatus*, Micky Lee	Infected leaf tissue	Rowan County, NC	2013
way-7-10	*P. cubensis*	Fragment analysis	*Citrullus lanatus*, Micky Lee	Infected leaf tissue	Haywood County, NC	2013
cle-11-12	*P. cubensis*	Both	*Momordica charantia*	Infected leaf tissue	Rowan County, NC	2013
way-11-7	*P. cubensis*	Fragment analysis	*Momordica charantia*	Infected leaf tissue	Haywood County, NC	2013
way-12-9	*P. cubensis*	Both	*Momordica balsamina*	Infected leaf tissue	Haywood County, NC	2013
way-12-6	*P. cubensis*	Fragment analysis	*Momordica balsamina*	Infected leaf tissue	Haywood County, NC	2013
14way-13-3a	*P. cubensis*	Fragment analysis	*Cucurbita foetidissima*	Infected leaf tissue	Haywood County, NC	2014
MSU-1	*P. cubensis*	Both	*Cucumis sativus*, cv Vlaspik	Sporangia	Homerville, Ohio, Provided by Brad Day (36)	2007
MSU2-B	*P. cubensis*	Both	*Cucumis sativus*, cv Vlaspik	Sporangia	MI, Provided by Mary Hausbeck	2013
sw003	*P. cubensis*	Fragment analysis	*Cucumis melo*	Sporangia	South Carolina, Provided by Peter Ojiambo	1982
NY10	*P. cubensis*	Fragment analysis	*Cucumis sativus*	Sporangia	Suffolk, New York, Provided by Christine Smart	2013
NY8	*P. cubensis*	Fragment analysis	*Cucumis melo*	Sporangia	Ontario, New York, Provided by Christine Smart	2013
NY60	*P. cubensis*	Fragment analysis	*Cucumis sativus*	Sporangia	Seneca, New York, Provided by Christine Smart	2013
SCD3	*P. cubensis*	Fragment analysis	*Cucurbita moschata*	Sporangia	South Carolina, Provided by Peter Ojiambo	2012
FL2013E1	*P. cubensis*	Fragment analysis	*Citrullus lanatus*	Sporangia	Florida, Provided by Peter Ojiambo	2013
CA081	*P. cubensis*	Fragment analysis	*Cucumis sativus*	Sporangia	California, Provided by Peter Ojiambo	2008
SL1010	*P. cubensis*	Fragment analysis	*Cucurbita pepo*	Sporangia	Israel, Provided by Yigal Cohen	2013
SANT2-5	*P. humuli*	Both	*Humulus lupulus*, cv Santiam	Sporangia	Henderson County, NC	2014
Cas5	*P. humuli*	Fragment analysis	*Humulus lupulus*, cv Cascade	Infected leaf tissue	Henderson County, NC	2014
HDM-501ba	*P. humuli*	Fragment analysis	*Humulus lupulus*, cv Pacific Gem	Sporangia	Oregon, Provided by David Gent	2012
HDM-499	*P. humuli*	Fragment analysis	*Humulus lupulus*, cv Pacific Gem	Sporangia	Oregon, Provided by David Gent	2013
hdm503ac	*P. humuli*	Fragment analysis	*Humulus lupulus*	Sporangia	Vermont, Provided by David Gent	2013
hdm481j-1	*P. humuli*	Fragment analysis	*Humulus lupulus,* feral	Sporangia	New York, Provided by David Gent	2011
hdm457 e3	*P. humuli*	Fragment analysis	*Humulus lupulus*	Sporangia	Oregon, Provided by David Gent	2011
hdm254	*P. humuli*	Fragment analysis	*Humulus lupulus*	Sporangia	Oregon, Provided by David Gent	2008
hdm257	*P. humuli*	Fragment analysis	*Humulus lupulus*	Sporangia	Oregon, Provided by David Gent	2008
hdm110-2	*P. humuli*	Fragment analysis	*Humulus lupulus*	Sporangia	Washington, Provided by David Gent	2006
hdm140	*P. humuli*	Fragment analysis	*Humulus lupulus*	Sporangia	Oregon, Provided by David Gent	2006
hdm482	*P. humuli*	Fragment analysis	*Humulus lupulus*	Sporangia	New York, Provided by David Gent	2011
hdm506cb	*P. humuli*	Fragment analysis	*Humulus lupulus*	Sporangia	New York, Provided by David Gent	2013
hdm484A	*P. humuli*	Fragment analysis	*Humulus lupulus*	Sporangia	Czech Republic, Provided by David Gent	2012
502aa	*P. humuli*	Fragment analysis	*Humulus lupulus*	Sporangia	Oregon, Provided by David Gent	2013
hdm490	*P. humuli*	Both	*Humulus lupulus*	Sporangia	Japan, Provided by David Gent	2012
hdm247	*P. humuli*	Fragment analysis	*Humulus lupulus*	Sporangia	Washington, Provided by David Gent	2008
hdm224	*P. humuli*	Fragment analysis	*Humulus lupulus*	Sporangia	Oregon, Provided by David Gent	2008
Gal	*P. humuli*	Fragment analysis	*Humulus lupulus,* cv Galena	Sporangia	Henderson County, NC	2014
Zeus	*P. humuli*	Fragment analysis	*Humulus lupulus,* cv Zeus	Sporangia	Henderson County, North Carolina	2014
Nug	*P. humuli*	Fragment analysis	*Humulus lupulus,* cv Nugget	Sporangia	Henderson County, North Carolina	2014
14wayhop14	*P. humuli*	Fragment analysis	*Humulus lupulus,* cv Pacific Gem	Infected leaf tissue	Haywood County, NC	2014

Results were individually analyzed and binned with the microsatellite plugin for the program Geneious version 8.1 (Kearse et al., 2012). On a given isolate fragment analysis run, peaks occurring one length of the repeat motif away from the peak with the highest signal and were less than 15% of the height of the larger peak were removed to decrease the risk of genotyping stutter peaks. Two alleles were assumed to be present at each locus because *Pseudoperonospora* spp., belonging to Oomycota, are diploid organisms. If one peak was observed at any given locus, homozygosity was assumed. Six of the 17 makers evaluated with fragment analysis proved to be monomorphic across the evaluated *P. cubensis* isolates and were removed from further analysis.

### Data analysis

Basic summary statistics were calculated for 11 reliable polymorphic primers across 38 *P. cubensis* isolates from diverse hosts, years, and locations, and 22 *P. humuli* isolates from diverse locations (Table 1). The R package Poppr version 2.2.0 was used to calculate descriptive population statistics such as the genotype accumulation curve, heterozygosity, and evenness, through the “gac” and “poppr” function (Nei, 1978; Pielou, 1975; Grünwald et al., 2003; Kamvar & Grunwald, 2014). To assess genotypic richness while accounting for sample size, a rarefaction curve was generated with the “vegan” package and “rarecurve” function. The dataset was clone-corrected with the “clonecorrect” function, and then, to determine if *P. cubensis* and *P. humuli* were sexual populations, the index of association was calculated with the “ia” function (Agapow & Burt, 2001). To evaluate the genetic distance between individuals, an UPGMA dendrogram based on Bruvo’s distance was created with 1,000 bootstrap replicates (R Core Team, 2016; Kamvar & Grunwald, 2014; Kamvar, Brooks & Grünwald, 2015).

## Results

### *In silico* identification and analysis of SSRs in predicted downy mildew transcriptomes

MISA analysis revealed that of the 23,522 *P. cubensis* sequences examined, 2,398 sequences contained microsatellites, with a total of 2,738 microsatellites identified. In *H. arabidopsidis*, 14,548 sequences were examined and 1,691 of the examined sequences contained microsatellites. A total of 2,119 microsatellites were identified in the *H. arabidopsidis* transcriptome. The number of SSR-containing sequences out of the total number of sequences examined were significantly different between species (*p* < 0.0002). A significant difference was also found between the percentage of total microsatellites identified out of the total number of sequences examined in *P. cubensis* (11.6%) and *H. arabidopsidis* (14.5%) (*P* < 0.0002). The total relative abundances of microsatellites in *P. cubensis* and *H. arabidopsidis* (101.79 and 152.01, respectively), show a greater difference between organisms compared to the percentage of SSR-containing sequences of the total number of sequences examined (10.2% and 11.6%, respectively). The total relative abundance (SSR/Mb) of microsatellites in *P. cubensis* and *H. arabidopsidis* revealed less of a difference compared to the total relative densities (bp/Mb) between the two species. *H. arabidopsidis* has a much higher density of microsatellites in the predicted transcriptome (2,322.89) compared to *P. cubensis* (1,421.66) (Table 2).

**Table 2:** Number and distribution of microsatellite in transcript sequences according to MISA.

	***P. cubensis***	***H. arabidopsidis***
Size of genome assembly (Mb)	64.33^a^	78.90^b^
Contig N50 (kbp)	3.96^a^	41.56^b^
Total number of sequences examined	23,522^a^	14,548^b^
Total size covered by examined sequences (Mb)	26.90	13.94
Total number of SSRs identified	2,738	2,119
Perfect	2,638 (96.4%)^c^	1,964 (92.7%)^c^
Compound	100 (3.7%)^c^	155 (7.3%)^c^
Number of SSR-containing sequences	2,398 (10.2%)^d^	1,691 (11.6%)^d^
Number of sequences containing more than one SSR	280 (1.2%)^d^	316 (2.2%)^d^
Total relative abundance (SSRs/Mb)^e^	101.79	152.01
Total relative density (bp/Mb)^f^	1421.66	2322.89

**Notes.**

a Data obtained from Savory et al. (2012a) and Savory et al. (2012b).

b Data obtained from Baxter et al. (2010).

c Percentage of total SSRs identified.

d Percentage of total number of sequences examined.

e Relative abundance is defined as the total number of SSRs per Mb of sequence analyzed.

f Relative density is defined as the total sequence length (bp) contributed by SSRs per Mb of DNA of total sequence analyzed.

A majority of the identified microsatellites in both *P. cubensis* and *H. arabidopsidis* sequences were perfect (96% and 93%, respectively), meaning there are no interrupting sequences within the chain of repeating units. Also, 1.2% of the sequences examined from the *P. cubensis* transcriptome contained more than one microsatellite, whereas 2.2% of the *H. arabidopsidis* sequences examined contained more than one microsatellite.

MISA analysis indicated that tri-nucleotide repeats were the most frequently occurring motif found in both transcriptomes (Table 3). This motif-type made up 61% of the total microsatellites from the *P. cubensis* transcriptome and 71% of the total microsatellites from the *H. arabidopsidis* transcriptome (Table 3). In both species, di-nucleotide repeats were the second most frequently occurring motif, followed by tetra-, hexa-, and penta-nucleotide repeats. There were significant differences found between the percentage of di- and tri-nucleotide repeats between species (*P* < 0.0002), but there were no significant differences found between the percentage of tetra-, penta-, and hexa-nucleotide microsatellites (*P* > 0.1). In terms of relative abundance, the same trend held with tri-nucleotide repeats having the highest value in both species, followed by di-, tetra-, hexa-, and then penta-nucleotide repeats. Although the relative density values for each motif group in *P. cubensis* also kept this trend, tetra-nucleotide repeats had a higher density than di-, hexa-, and penta-nucleotide repeats in *H. arabidopsidis*. Also, in *H. arabidopsidis* higher relative abundance and relative density values were seen for each motif group except di-nucleotide repeats, in which *P. cubensis* held higher values (Table 3). It should also be noted that in the *H. arabidopsidis* transcriptome, there was only one monomer identified that met the specification stated in the MISA script of 20 repeating units. There were no monomers identified in the *P. cubensis* transcriptome.

**Table 3:** Percentage, relative abundance, and relative density of microsatellites in downy mildew transcripts.

	**Motif length**	**Count**	**Percentage**^a^	**Relative abundance**^b^	**Relative density**^c^
***P. cubensis***	di	563	20.56%	20.93	205.44
	tri	1,675	61.18%	62.27	732.99
	tetra	305	11.14%	11.34	133.10
	penta	47	1.72%	1.75	26.02
	hexa	148	5.41%	5.50	89.45
***H. arabidopsidis***	di	252	11.89%	18.08	172.74
	tri	1,511	71.31%	108.40	1207.53
	tetra	221	10.43%	15.85	178.19
	penta	29	1.37%	2.08	26.54
	hexa	105	4.96%	7.53	114.06

**Notes.**

a Percentage was calculated for each organism on the basis of the corresponding total SSRs count.

b Relative abundance is defined as the total number of SSRs per Mb of sequence analyzed.

c Relative density is defined as the total sequence length (bp) contributed by SSRs per Mb of DNA of total sequence analyzed.

**Figure 1:**
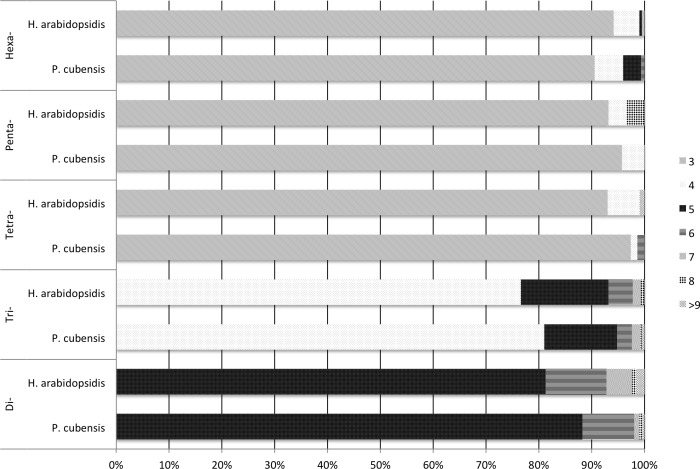
Frequency of number of repeats by motif-type and source transcriptome.

Both transcriptomes had the same repeating sequences occur with high frequency. For example, the motif AGC/CTG was the most commonly occurring motif in both transcriptomes, being the repeating sequence in 451 of the microsatellites identified in the in the *P. cubensis* transcriptome and 495 of the microsatellites identified in the *H. arabidopsidis* transcriptome. Eleven out of the fifteen most common motif sequences were the same for both *P. cubensis* and *H. arabidopsidis* (Table 4). A majority of the microsatellites identified in each motif-type group fell toward the lower bound of the repeat range set for microsatellite identification using MISA (Fig. 1). MISA was to identify repeating sequences that exceeded five repeating motifs for di- nucleotide repeats, four repeating motifs for tri-nucleotide repeats, and three repeating units for tetra-, penta-, and hexa-nucleotide repeats. In both species, over 90% of tetra-, penta-, and hexa-nucleotide repeat microsatellites were made up of three repeating units. A higher percentage of longer chains of repeating units occurred in di- and tri- nucleotide repeats. For example, tri-nucleotide repeat microsatellites with more than four repeating units made up 19% of the tri-nucleotide repeat microsatellites in *P. cubensis* and approximately 23% of the tri-nucleotide repeat microsatellites in *H. arabidopsidis* (Fig. 1).

**Table 4:** Most common repeat motifs identified from perfect and compound microsatellites in two downy mildew transcriptomes.

***P. cubensis***		***H. arabidopsidis***	
**Motif**	**Count**	**Motif**	**Count**
AGC/CTG	451	AGC/CTG	495
CG/CG	400	AAG/CTT	330
AAG/CTT	359	ACG/CGT	227
CCG/CGG	238	AGG/CCT	149
ACG/CGT	172	AG/CT	119
ACC/GGT	164	ATC/ATG	107
AGG/CCT	104	ACC/GGT	82
ATC/ATG	104	AC/GT	76
AG/CT	87	AAC/GTT	55
AC/GT	74	CG/CG	48
AAC/GTT	69	CCG/CGG	40
CCGG/CCGG	33	ACAG/CTGT	29
CCCG/CGGG	32	ACT/AGT	24
AGCC/CTGG	28	AAGG/CCTT	19
AAGC/CTTG	27	AGCG/CGCT	17

### Primer design and lab validation

Primers were successfully designed by Primer3 to amplify 2,088 microsatellites out of the 2,738 microsatellites identified by MISA in the transcriptome of *P. cubensis*. A majority of these primers (97%) were for amplification of perfect microsatellites, meaning there are no sequences interrupting the repeating motif; however, 3% of the primers are predicted to amplify compound microsatellites, where non-SSR base pairs may be found within the repeating motif sequence. Of the 2,088 primers designed, 417 were di-nucleotide repeats, 1,125 were tri-nucleotide repeats, 248 were tetra-nucleotide repeats, 40 were penta-nucleotide repeats, and 91 were hexa-nucleotide repeats.

**Figure 2:**
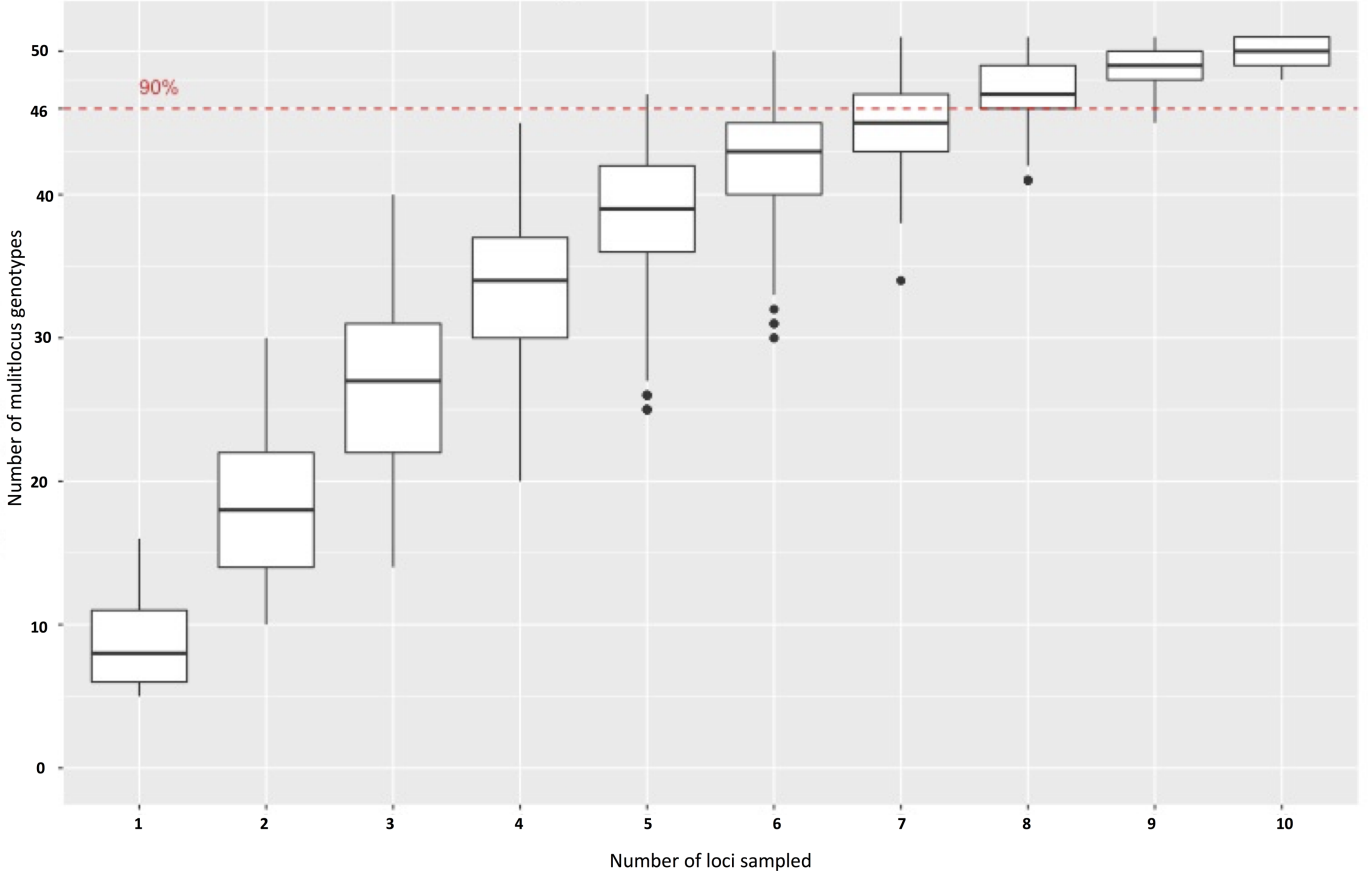
A genotype accumulation curve for 51 isolates of *P. cubensis* and *P. humuli* across 11 loci. The dashed line marks the set threshold of when 90% of the observed MLGs can be detected. This occurs when 7–8 markers are used.

Experimental validation of 100 primer sets with gel electrophoresis (Table S1) revealed that 92% of the selected markers produced a product across the *P. cubensis* isolates used, and 90% of the 92 primer pairs that produced a product were the size predicted by Primer3 (83 total). The electrophoresis results also revealed a majority of the primers showed significant species transferability. Of the 85 primers that produced a product in more than just the positive control isolate (MSU-1), only one primer set, SSR94, did not produce a product in the *P. humuli* isolates screened. Of the 92 primers that produced a PCR product of the Primer3 prediction, seven primers only amplified the isolate that was used to sequence the *P. cubensis* genome (MSU-1) (Savory et al., 2012a; Savory et al., 2012b;). Of the initial 100 primer sets selected to be experimentally validated with gel electrophoresis, 17 primer sets had consistent amplification and appeared to be polymorphic across *P. cubensis* isolates. When the 17 primers were applied to a larger panel of *P. cubensis* isolates (*n* = 38), only 11 primer sets were identified to consistently amplify loci with polymorphic alleles within *P. cubensis* (Table 5). These 11 markers were determined to be polymorphic via the “informloci” function in poppr. When the loci were sampled 1,000 times without replacement, the genotype accumulation curve demonstrated that 7–8 markers were necessary to discriminate between 90% of the multilocus genotypes (Fig. 2). Five of these 11 polymorphic primers were tri-nucleotide repeats and four were hexa-nucleotide repeats, initially selected based on predicted number of repeats. The final two polymorphic primers were selected because they were predicted to be located in function-associated genes. These two markers, SSR97 and SSR92, were identified in putative Crinkler family proteins.

**Table 5:** Statistics of polymorphic primers for *P. cubensis* and *P. humuli*.

**SSR name**	**Forward primer**	**Reverse primer**	**Gene annotation**	**Motif**	**Allele size range**	**No. of alleles**		**Heterozygosity**^a^		**Evenness**^b^	
						*P. cubensis*	*P. humuli*	*P. cubensis*	*P. humuli*	*P. cubensis*	*P. humuli*
SSR79	TGGCATGGC TTCGTACATGT	TAGTGGTGA GGAGGGGTCTG	Tankyrase 2	(TCT)7	430–448	2	2	0.39	0.24	0.81	0.63
SSR85	GGAGGAGGA GGAGGAGGAAG	TCAACGTCG GGATCTTGACG	Digestive organ expansion factor	(AGA)7	285–390	6	5	0.60	0.18	0.72	0.38
SSR97	TGTTTCCGG TGAAGATCGCA	GCTTCCACGA TGAACGCATC	Crinkler (CRN) family protein	(GA)5	241–253	5	3	0.57	0.59	0.79	0.88
SSR102	CAAAAAGCG CGATATCGGCA	CCCAACC ACGTCTTCTTCGA	Crinkler (CRN) family protein	(AGA)4	288–309	7	4	0.70	0.61	0.69	0.80
SSR57	GACAAAAA CGTGGACACCCG	TGGACCTT TTCCCCCATTGG	ATP-binding Cassette (ABC) Superfamily	(GGCGGT)4	230–284	5	2	0.26	0.44	0.44	0.88
SSR66	AGCGTCGTT CACCAAGATGT	CAGTGTCGTT GGCTGTTTCG	Type II (General) Secretory Pathway (IISP) Family	(TGGAGG)3	243-303	6	4	0.74	0.33	0.85	0.49
SSR34	AGGTGCAA GGTCTGATGACG	TCCTTCACT CTCCCTGTCGT	TIMELESS interacting protein	(AGA)7	162-198	3	2	0.26	0.46	0.52	0.91
SSR88	CAAATGCC CATGGGAATGCC	ACTCATCT GCGCGATCTGAG	Conserved gene of unknown function	(AATGCA)3	118–130	3	2	0.51	0.50	0.80	0.98
SSR29	GGAAGAAG AGGGCGACACAA	GATCTATG CTGGGTGCTGCT	AP-1 complex subunit beta	(CAA)8	122–176	7	3	0.35	0.21	0.42	0.49
SSR16	TCAGCCTT CTAATGCCCAGC	GTTGCTGT TGTTGCTGCTGT	Multiple banded antigen	(CAACAG)6	236–278	4	2	0.66	0.10	0.88	0.48
SSR1	TAGCTGCT GTGGATGTGACG	TACTTTCTC TGGGCAGCTGC	Conserved gene of unknown function	(AAG)12	275–341	9	3	0.75	0.49	0.68	0.73
Mean						5.18	2.91	0.53	0.38	0.69	0.70

**Notes.**

a Heterozygosity is Nei (1978) gene diversity.

b Evenness is a measure of the distribution of MLGs within the isolates (Pielou, 1975; Grünwald et al., 2003).

### Marker characterization and summary statistics

**Figure 3:**
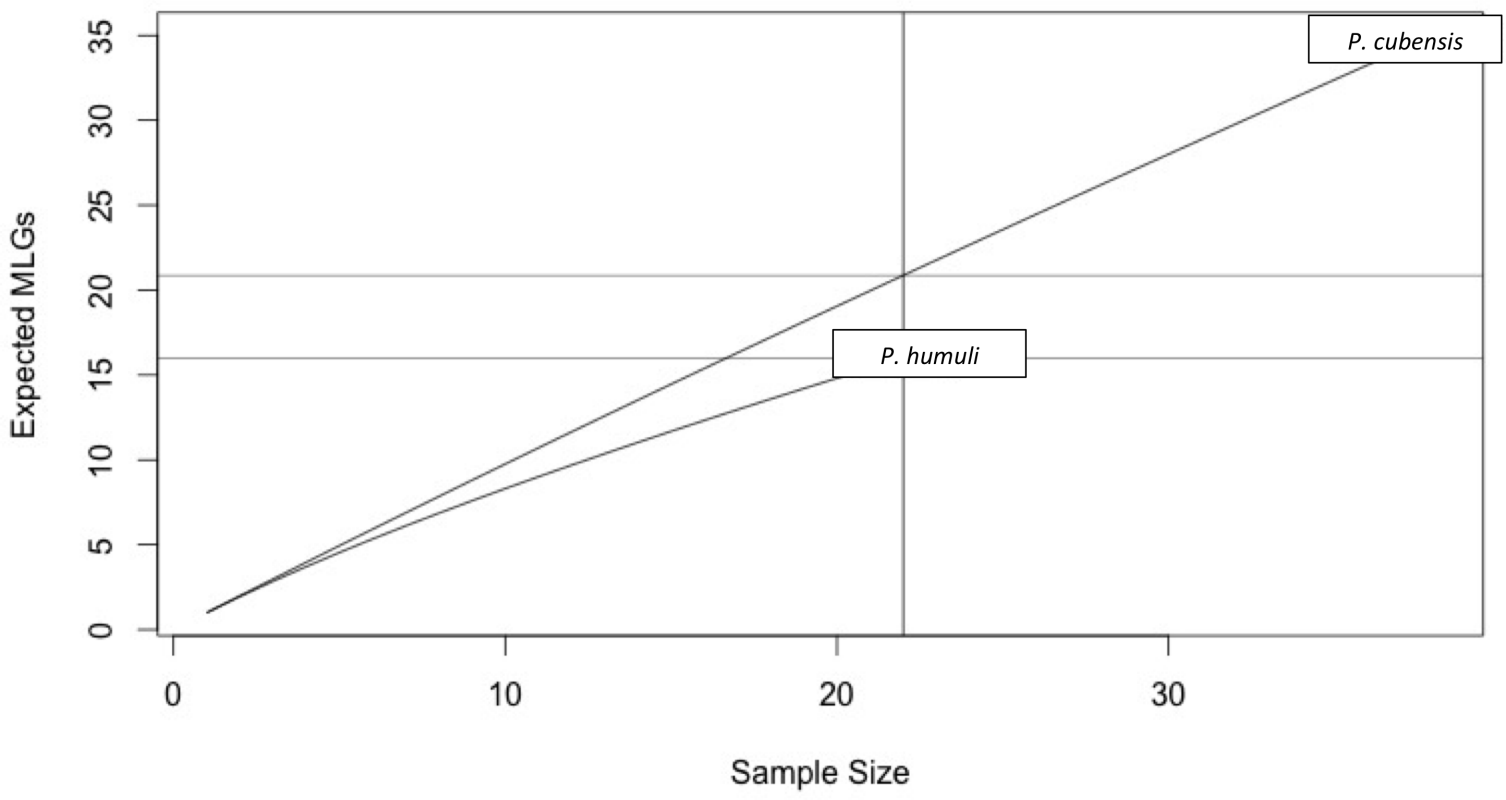
Multi Locus Genotypes for *Pseudoperonospora* isolates. A rarefaction curve demonstrating *P. cubensis* is expected to have 20.87 MLGs and *P. humuli* is expected to have 16 MLGs at the largest, shared sample size (*n* = 16).

When these 11 primers were applied to a diverse panel of *P. cubensis* isolates from all major commercial hosts and three non-commercial cucurbits spanning several years and geographic locations (*n* = 38) and *P. humuli* isolates from diverse geographic regions (*n* = 22), descriptive population statistics could be determined. Over eleven loci, 89 alleles were found in the isolates evaluated. Overall, more alleles were found at each locus across *P. cubensis* isolates, with the exception of SSR79, in which two alleles were found across the set of isolates for each species. The number of alleles per loci ranged from two to nine, with an average allele diversity of 5.18 alleles per locus in *P. cubensis* and 2.91 alleles per locus in *P. humuli*. Heterozygosity across all isolates had a mean value of 0.45, with the mean heterozygosity being 0.53 in *P. cubensis* and 0.38 in *P. humuli.* This higher genotypic richness seen in *P. cubensis* isolates was further confirmed by generating a rarefaction curve to account for differences in sample size (Fig. 3). The evenness of alleles at each locus ranged from 0.38 to 0.98, with the mean evenness of 0.69 and 0.70 in *P. cubensis* and *P. humuli*, respectively, showing a similar moderate distribution of multi locus genotypes (MLGs) in both species (Table 5).

**Figure 4:**
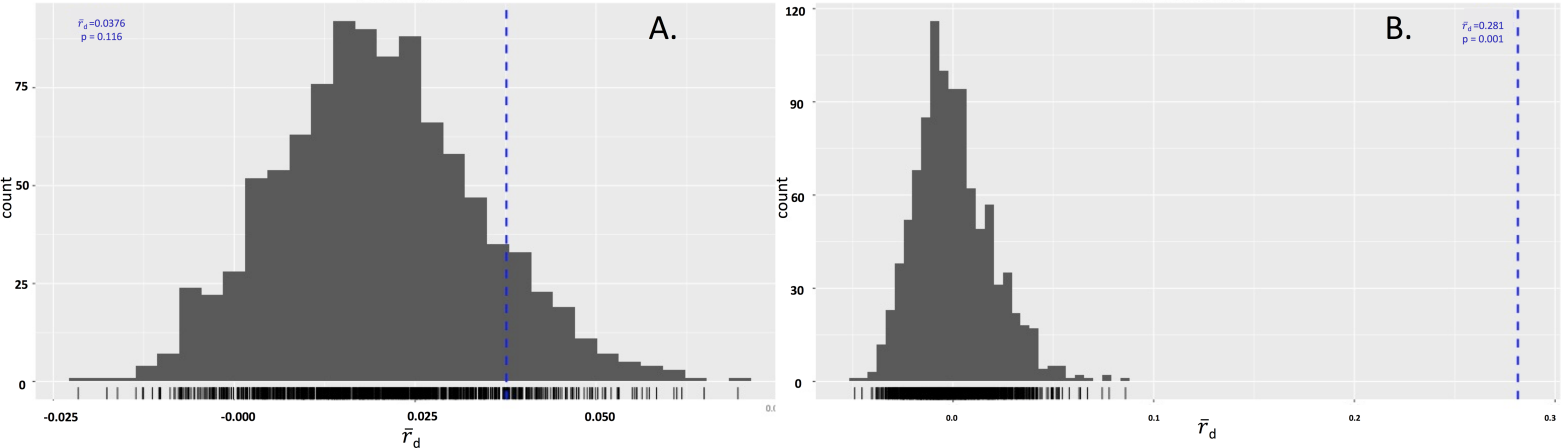
Index of association in *Pseudoperonospora* isolates calculated from 999 resamplings. (A) The distribution of *I*_*A*_ for *P. cubensis* isolates showing a failure to reject the null hypothesis of sexual reproduction. (B) The distribution of *I*_*A*_ for *P. humuli* showing a rejection of the null hypothesis, thus suggesting a clonal population.

The isolates were also tested to determine if either species were in linkage disequilibrium. In *P. cubensis* isolates, the ${\overline{r}}_{\mathrm{D}}$ value was 0.04, which lies within the distribution expected under linkage. The *p* value of 0.116 does not reject the null hypothesis that the alleles seen across the 11 loci are not linked, suggesting the *P. cubensis* isolates are sexually reproducing. On the other hand, the ${\overline{r}}_{\mathrm{D}}$ value for the *P. humuli* isolates was 0.28, which falls outside of the distribution expected under linkage. The *p* value of 0.001 supports the rejection of the null hypothesis that the alleles seen across the 11 loci are not linked, suggesting the *P. humuli* isolates are clonal (Fig. 4).

**Figure 5:**
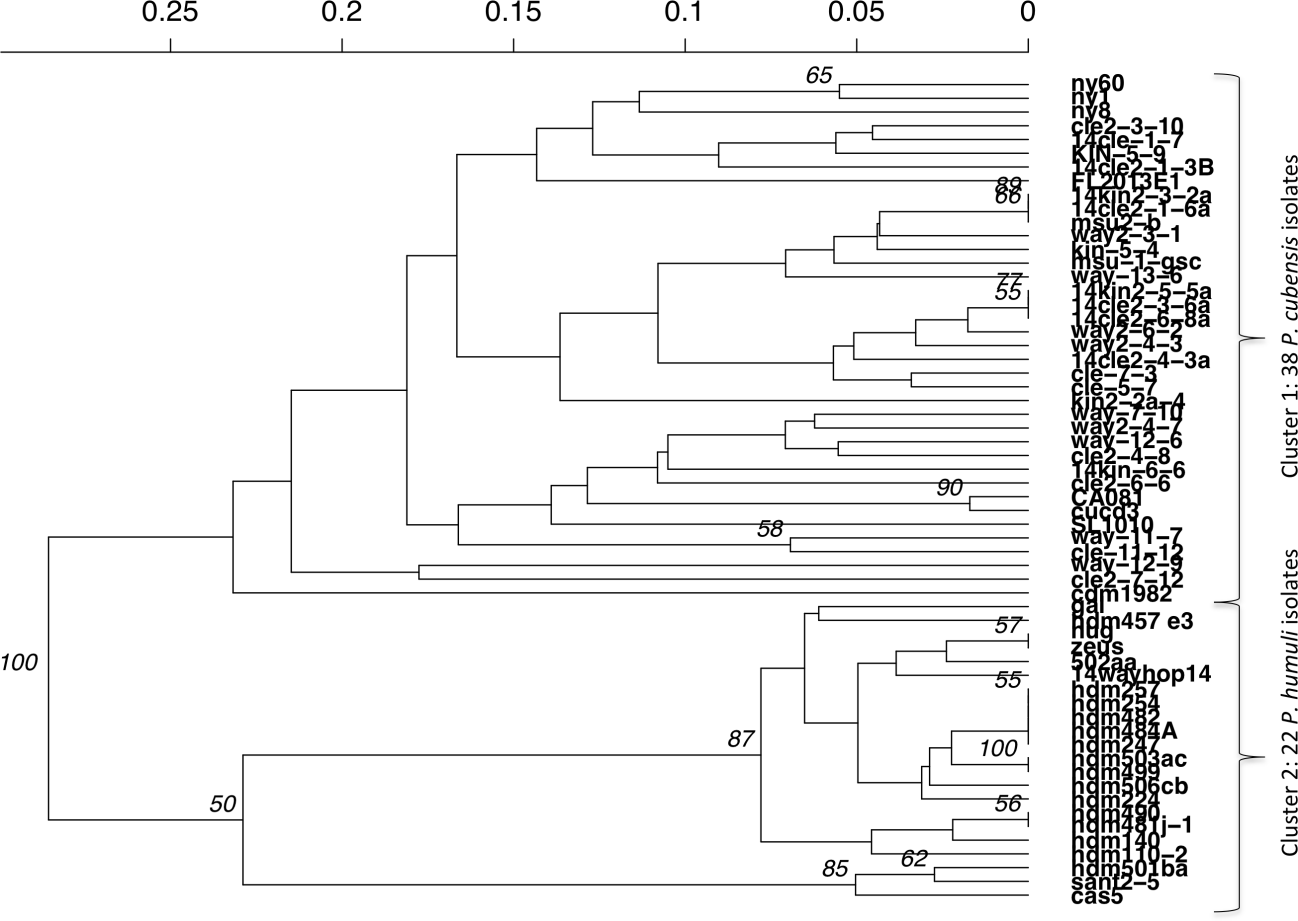
UPGMA dendrogram of Bruvo’s distance between individuals of *P. cubensis* and *P. humuli*. Analysis implemented 1,000 bootstrap replications.

Distinct differences were seen between *P. cubensis* and *P. humuli* isolates when an UPGMA dendrogram was generated for Bruvo’s distance between individuals. Two clear groups could be seen based on comparison between individuals without taking population into consideration and is supported with 100% confidence, as the analysis had 1,000 bootstrap replications. Cluster 1 as seen in Fig. 5 includes only *P. cubensis* isolates. *P. humuli* isolates, on the other hand, group together in Cluster 2. There is some support for distinct clusters within the *P. humuli* group, the smaller of which contain three *P. humuli* isolates, Cas, Sant2-5, and Hdm501ba. These isolates are from North Carolina and Oregon, but so are several *P. humuli* isolates that fall into the larger *P. humuli* cluster. Cluster1 comprises of *P. cubensis* isolates, with no clear trend or support of sub-clustering by host or location. However, it is clear the diversity within *P. cubensis* isolates is much more varied and complex compared to that of *P. humuli*. Trends may be become apparent when a larger dataset with more isolates per host and location is evaluated.

## Discussion

Evolutionarily, microsatellite distribution throughout the genome is of interest because of their frequency throughout most eukaryotic genomes and their high mutation rates. The number of repeating units at a given loci and the frequencies of each resulting allele offer insight into evolutionary events that shaped the current population (Ellegren, 2004; Schlötterer, 2000; Oliveira et al., 2006; Tóth, Gáspári & Jurka, 2000). Although the exact role microsatellites have within the genome is not well understood, studies have suggested possible contribution to genome organization and stability (Li et al., 2004). Repeating sequences with high mutation rates located within genes may be of particular interest because they may have a role in functionality (Li et al., 2004; Oliveira et al., 2006). The most well studied examples of effects of microsatellites in coding regions arise from studies on human diseases. In particular, several neurological diseases are associated with unstable microsatellite repeats (Orr & Zoghbi, 2007; Brouwer, Willemsen & Oostra, 2009). As of now, it is uncertain whether that possible effect on functionality has led to isolate/population variability in pathogenicity or virulence. Several microsatellites were located within predicted pathogenicity-related genes, some of which were examined in this study. Future studies may wish to investigate possible involvement of microsatellites in pathogen virulence.

Interesting comparisons can be made between the predicted transcriptomes of *P. cubensis* and *H. arabidopsidis*, two downy mildew pathogens with publically available genomes. The percentage of microsatellites in examined sequences was significantly different between *P. cubensis* and *H. arabidopsidis*, being 12% and 15%, respectively. Although a larger portion of sequences originating from the *P. cubensis* genome was examined compared to those of the *H. arabidopsidis* genome, microsatellites were more abundant in the *H. arabidopsidis* sequences. This may be attributed to the differences in the number of sequences examined relative to the genome size and quality between the genome assemblies (Savory et al., 2012a; Savory et al., 2012b; Baxter et al., 2010). *P. cubensis* had more sequences examined (23,522) for a smaller assembled genome (64 Mb) but low Contig N50 value of 3.96 kbp. *H. arabidopsidis* on the other hand had less sequences examined (14,548) for a larger assembled genome (79 Mb) but with a Contig N50 value of 41.56, considerably higher than that of the *P. cubensis* genome (Table 2). Relative abundance (SSR/Mb) and relative density (bp/Mb) values may be more informative because these values account for the differences in length of examined sequences between species. Relative abundance is the number of microsatellites observed per Megabase of sequence examined, and relative density is the total length of sequence contributed by microsatellites per Megabase of sequence examined. *H. arabidopsidis* had higher relative abundance of microsatellites and higher relative density of microsatellites compared to that of *P. cubensis*.

These differences may also be influenced by the separate methodologies in whole genome sequencing and assembly used in each project. The genome for *H. arabidopsidis* merges data from Sanger shotgun sequencing and Illumina sequencing and annotations were performed ab initio and with BLASTX to the NCBI database (Baxter et al., 2010). The *P. cubensis* genome was generated via Illumina sequencing and annotated with MAKER using oomycete genome data (Savory et al., 2012b). Furthermore, the methods used to generate the transcriptome files used as our input for MISA in our study also differed between *H. arabidposidis* and *P. cubensis.* The transcriptome file from *H. arabidopsidis* was created using Sanger EST sequencing and 454 cDNA sequencing, and transcripts were extracted from the *P. cubensis* genome using predicted gene coordinates from gene annotation files. With few downy mildew pathogen genomes sequenced and the issues associated with assembly of genomes of obligate pathogens, it is difficult to know how many genes are expected in each species. The availability of a diverse panel of downy mildew pathogen genomes could further answer these questions (Withers et al., 2014; Sharma et al., 2015; Derevnina et al., 2015).

Other than differences in microsatellite abundance, many frequency and distribution trends were similar between the two downy mildew pathogens. Tri-nucleotide repeats appear in the greatest abundance for both species, a common trend in oomycete pathogens (Garnica et al., 2006; Biasi, Martin & Schena, 2015). This is to be expected in coding regions, as tri-nucleotide repeats would be less likely to cause frame-shift mutations, therefore it is thought that size of the repeat unit is subject to selection pressure (Li et al., 2004; Selkoe & Toonen, 2006; Tóth, Gáspári & Jurka, 2000; Metzgar, Bytof & Wills, 2000).

Many of the microsatellites identified fell close to the minimum size set in the MISA parameters. Between 77% and 99% of the microsatellites identified for each motif-type had the lowest possible number of repeating units as specified by the MISA script (Fig. 1). This seems to be a trend observed in other surveys of microsatellites in expressed regions (Cai et al., 2013; Liu et al., 2013; Coulibaly et al., 2005). Studies have shown that the number of repeating bases in microsatellites located in expressed regions tend to be low (Li et al., 2004; Garnica et al., 2006). In microsatellite evolution, the number of repeating units tends to correlate with mutation rate (Schlötterer, 2000).

The program Primer3 designed primers for 76% of the microsatellites identified by MISA. The number of primers developed from the *P. cubensis* predicted transcriptome by Primer3 for each repeat-type group (tri-, tetra-, penta-, and hexa-nucleotide repeats) followed the same trend as the identified microsatellites predicted by MISA. This suggests a lack of bias in the likelihood of primer design for a particular motif type.

When primers designed by Primer3 were validated via PCR reactions and gel electrophoresis, it was found that the percentage of primers corresponding to the Primer3 output was high (92%). This suggests that the *P. cubensis* predicted transcriptome is a reliable source of *in silico* marker identification (Savory et al., 2012a; Baxter et al., 2010). Interestingly, any deviation from the Primer3 predicted product size greater than 25 base pairs was mainly due to a larger product size, and in most cases, the validated product size was two times the size of the predicted product (Table S1). Where small deviations between predicted and observed product size may be due to limited resolution in gel electrophoresis, these greater differences may arise because of the difficulty in capturing true length of repeating regions during genome assembly (Treangen & Steven, 2012).

Over the years, there have been several hypotheses concerning the relationship between these two *Pseudoperonospora* species (Hadziabdic et al., 2013; Mitchell et al., 2011; Choi, Hong & Shin, 2005; Sarris et al., 2009; Runge, Choi & Thines, 2011). High morphological and sequence similarity have made these two economically important pathogens difficult to discriminate. This becomes especially problematic in regions where both cucurbit crops and hop yards are prevalent and spore traps are used to scout for disease (Gent et al., 2009). New molecular tools that can differentiate between these *Pseudoperonospora* may serve as a much needed diagnostic tool (Withers et al., 2014). However, the examined microsatellites revealed that although no MLGs were common between the two species, alleles were shared between the *P. cubensis* and *P. humuli* isolates. Furthermore, Bruvo’s genetic distance showed strong support for *P. cubensis* isolates clustering separately from *P. humuli* isolates in an UPGMA dendrogram. This information may also be useful in future investigations on gene flow between these species, speciation in *Pseudoperonospora*, and other evolutionary relationships.

When the identified polymorphic markers were applied to a larger, diverse panel of *P. cubensis* isolates, it was found that the average heterozygosity across *P. cubensis* isolates was 0.53. In a recent study, researchers from Michigan used microsatellites mined from the *P. cubensis* predicted transcriptome to evaluate *P. cubensis* populations across relatively small geographic regions and over the course of one growing season. The population analyzed in their study had an average genetic diversity of 0.69 and were able to use their markers to detect structure and determine variation in populations from different counties in Michigan and Ontario, as well as variation between isolates collected from different time points (Naegele et al., 2014; Naegele et al., 2016). Markers used in the Michigan study were selected for their polymorphism among isolates in the Great Lakes region, whereas the markers in the current study were selected for polymorphism among isolates primarily from North Carolina. Differences in genetic diversity values between this study and ours are likely due to the primers selected. However, the results of the Michigan study suggest that if the markers identified in our study were applied to a much larger number of isolates, structure could also be detected in other distinct geographic region in the southeastern US and also over the course of a growing season.

The informative nature of these microsatellites is particularly promising in that more population studies are needed to understand the finer details of *P. cubensis* populations in the US. In particular, a 2012 study conducted by Quesada-Ocampo, Granke & Olsen (2012) used sequence data to survey *P. cubensis* on a global scale and detected some geographic and host differentiation, with certain genetic clusters occurring more frequently in certain continents or hosts. It also found high genetic diversity in certain regions of the United States, particularly in Georgia, North Carolina, and Indiana (Quesada-Ocampo, Granke & Olsen, 2012). However, this previous study had limited sampling in certain regions. Also, using more genetic regions might capture more diversity than was seen in the results of this study. If markers were used that could detect differences in a large sampling of isolates from a distinct geographic location, particularly a location with high genetic diversity, details on population structure on a finer scale could be determined.

As Europe recently experienced a shift in *P. cubensis* virulence (Lebeda & Cohen, 2011), similarly to what the US experienced in 2004–2005 (Holmes et al., 2015), researchers used AFLPs to study *P. cubensis* populations across Europe and found two main clades separating Central and Western Europe from the Mediterranean (Sarris et al., 2009). A later study using ISSRs and SRAPs was able to determine that isolates from Israel are distinct from groups in the Czech Republic and Turkey. Although informative on a geographic level, the studies had hoped to find differentiation based on host of origin, mating types, and pathotypes, and called for the importance of a different molecular marker (Polat et al., 2014). The use of microsatellites developed in our study may be another cost-effective tool that can aid our understanding of *P. cubensis* diversity.

Furthermore, the cross-species transferability of the identified markers provides potential for further exploration of the relationships between *Pseudoperonospora* species. A majority of the primers designed *in silico* from the *P. cubensis* draft genome had successful amplification in diverse *P. humuli* isolates. The markers showed higher genetic diversity in *P. cubensis* isolates compared to *P. humuli* isolates, although genotypic evenness was similar between the two species. This appears to be consistent with the findings of population studies on the two *Pseudoperonospora* species (Quesada-Ocampo, Granke & Olsen, 2012; Naegele et al., 2016; Chee et al., 2006). These isolates, when genotyped along with *P. cubensis* isolates, showed distinct differentiation between species. Analyses of index of association also showed support for different lifestyles of these species, with indications of *P. cubensis* undergoing sexual reproduction and *P. humuli* being clonal. This is also supported by previous findings of the occurrence of mating types within *P. cubensis* and claims of homothallic lifestyle of *P. humuli* (Cohen et al., 2015; Cohen & Rubin, 2012). It should be noted that in laboratory settings, Runge & Thines (2012) demonstrated that there is some level of cross-infectivity of *P. cubensis* on *Humulus lupulus* and *P. humuli* on *Cucumis sativus*. The strains of *P. cubensis* and *P. humuli* used in the study were also able to reproduce on *Bryonia dioica*, also in laboratory settings (Runge & Thines, 2012). Further examinations of the relationship between these sister species should consider this factor.

Overall, the publically available predicted transcriptomes of downy mildews offer a wealth of information to contribute insight to downy mildew genomics as well as avenues for application in addressing questions about the population biology of downy mildew pathogens. Major similarities in microsatellite frequency and distribution in predicted genes were observed between *H. arabidopsidis* and *P. cubensis* despite differences in life cycle and host range. Primer3 was able to provide a substantial source of markers from *P. cubensis* transcripts that had high fidelity to predicted products. A majority of the markers were transferable to another economically important *Pseudoperonospora* species, and could potentially be transferable to other genera of downy mildew pathogens. Finally, many of the markers identified were able to detect diversity within a small panel of *P. cubensis* isolates. Analysis of local populations using molecular markers is essential when resolving specific aspects of pathogen dispersal through a region. Ultimately, these markers can provide the insight necessary to optimize disease management strategies for this devastating pathogen.

## Supplemental Information

10.7717/peerj.3266/supp-1 Fig. S1 Predicted transcriptome of *Pseudoperonospora cubensis* generated from the draft genome This file is the predicted transcriptome file for *Pseudoperonospora cubensis*. This dataset was used to mine for microsatellites, or simple sequence repeats (SSRs).

10.7717/peerj.3266/supp-2 Table S1 Microsatellite markers mined from *Pseudoperonospora cubensis* transcriptome and screened against *P. cubensis* and *P. humuli* isolates

## Additional Information and Declarations

### Data Availability

The following information was supplied regarding data availability: The raw data has been supplied as Supplementary Files.
